# Understanding the Impact of Green Human Resource Management Practices and Dynamic Sustainable Capabilities on Corporate Sustainable Performance: Evidence From the Manufacturing Sector

**DOI:** 10.3389/fpsyg.2022.844488

**Published:** 2022-06-29

**Authors:** Mahvish Kanwal Khaskhely, Sarah Wali Qazi, Naveed R. Khan, Tooba Hashmi, Asma Abdul Rahim Chang

**Affiliations:** ^1^Institute of Science, Technology and Development, Mehran University of Engineering and Technology (MUET), Jamshoro, Pakistan; ^2^Department of Management Science, Shaheed Zulfiqar Ali Bhutto Institute of Science and Technology, Karachi, Pakistan; ^3^Department of Management Studies, Bahria Business School, Bahria University, Karachi, Pakistan; ^4^Department of Management Studies, Faculty of Business and Management, UCSI University, Kuala Lumpur, Malaysia; ^5^Department of Management Science, Mohammad Ali Jinnah University, Karachi, Pakistan

**Keywords:** green human resource management, dynamic sustainable capabilities, manufacturing sector, green recruitment and selection, green pay and reward, corporate sustainable performance, social equity, ecological conservation

## Abstract

Pakistan ranks as the eighth most vulnerable country on the 2021 global climate change vulnerability index. Partially, this perilous position is attributed to unsustainable practices in the large-scale manufacturing sector since its contribution to carbon emission is among the highest in the economy. These serious environmental challenges impede the attainment of sustainable development goals that concern responsible consumption and production. In manufacturing organizations, there are an ongoing debate regarding sustainable human resource management (HRM) determinants, which can promote sustainable performance. In this regard, green human resource management (GHRM) practices and dynamic sustainable capabilities are significant components as they have a unique role in transforming corporations into sustainable organizations. However, there is a dearth of evidence regarding the impact of individual GHRM practices, such as green recruitment and selection, green pay and reward, and sustainable capabilities like monitoring and re-configuration, in improving the corporate environmental and social performance. Hence, an empirical investigation regarding the association among these macro-level components with the corporate environmental and social performance through partial least squares structural equation modeling (PLS-SEM) is conducted. The findings inferred from 396 employees affiliated with six large-scale industries substantiate the main hypotheses of this study. It is empirically confirmed that GHRM and dynamic sustainable capabilities significantly and positively impact corporate sustainable performance. This research contributes to the literature by employing dynamic capabilities approach and a dynamic resource-based view (RBV) to explicate how corporations can benefit from the interplay of sustainable capabilities and GHRM functions. Hence, in the absence of a significant predictive model, this research is the first of its kind to isolate macro-level antecedents of sustainable HRM to find their impact on corporate sustainable performance in a developing country context. The study recommends that the management should prioritize the acquisition of monitoring capabilities and hiring environmentally conscious employees to achieve social equity and ecological conservation goals.

## Introduction

Globally, the large-scale manufacturing sector creates an enormous amount of waste, exploitation of natural resources, overconsumption of energy, and unsustainable workplace practices (Abdul-Rashid et al., 2017). This case is especially true for developing countries; despite exhibiting lucrative market growth potential, they are highly vulnerable to environmental and social exploitations and crises (Masud et al., 2018). Pakistan is no exception since it is the 5th most populous country in the world with 220 million inhabitants (Mukhtar, 2020; The World Bank, 2020). The Global Climate Risk Index 2021 ranks it as the 8th worst stricken country in the world (Eckstein et al., 2021). This index analyses and ranks countries and regions based on the extent to which they are affected by climate-related extremities like heatwaves and floods. The industrial or manufacturing sector is the largest contributor to the GDP of this developing country (Sarstedt et al., 2019). Consequently, it is one of the primary sources of carbon emission (approximately 21%) in the country after the agricultural sector (Tanveer et al., 2021). According to Dissanayake et al. (2016) the manufacturing sector is the major cause of unmanaged industrial waste, unsustainable workplaces, and Human Resource practices. Therefore, this sector and its sustainability issues lend themselves for further investigation in this research.

Moreover, many scholars suggest that the corporate solution to sustainability issues can be materialized through sustainable ecological and social performance. Ecological performance is the expression of the corporate commitment to conserve the environment through measurable, operational indicators concerning ecological care (Roscoe et al., 2019; Haldorai et al., 2022). Whereas, corporate social performance is determined through improved health and safety of internal and external stakeholders, creation of job opportunities, and reducing the negative impact of the organization on the community (Mousa and Othman, 2020). Collectively, corporate sustainable performance is essential for survival, ecological conservation, and improving the human quality of life. It enhances employee productivity and reduces the time and costs of hiring and attrition (Tang et al., 2018). Further, it facilities the corporations in creating and maintaining their competitive edge in the global market apart from ensuring survival and conservation of the eco-system and societal wellbeing (Orji, 2019). Therefore, both practitioners and academicians need to have a clear understanding of the factors affecting corporate sustainability performance (Jamal et al., 2021).

Furthermore, corporate sustainability performance is dependent on the management and employees’ green practices like GHRM and dynamic capabilities (Schaltegger and Burritt, 2018). Green HRM refers to sustainable HRM practices that have an ecological impact on the organization. It is a fundamental component of corporate sustainable strategy, promoting across the board employee green behaviors and ecological and social performance (Hameed et al., 2020; Singh et al., 2020). It consists of key practices like green recruitment and selection and, green pay and reward. The role of green human resource management (GHRM) is crucial in the development of environment-friendly norms and practices within organizations and ultimately leads to improved corporate sustainable performance (Yong et al., 2020).

Apart from GrHRM, other antecedents of a sustainable organization include dynamic sustainable capabilities. Organizational capabilities contribute to implementing corporate sustainability strategies. They enhance corporate sustainability performance as they constitute an important part of a firm’s business strategy in many industries (Shahzad et al., 2020). Dynamic sustainable capabilities are special kinds of corporate capabilities that facilitate organizations to systematically sense, seize, monitor, and configure sustainable development opportunities to achieve superior performance in the ecological and social domains (Wu et al., 2013). The discussion of organizational capabilities in the corporate sustainability literature is limited. Therefore, based on its relevance in dealing with external and internal dynamism and its representation in dynamic resource-based theory, its inclusion is made in the current study.

Furthermore, in the developing country context, despite being the largest contributor to GDP, export, and employment creation after agriculture, there is a dearth of literature regarding corporate sustainability in the manufacturing sector (Bin Saeed et al., 2018; Kim et al., 2019). Corporate sustainable practices are crucial for the sector to successfully mitigate the ecological crisis and promote safer and more humane workplaces (Summers et al., 2014; Koho et al., 2015; Shang et al., 2020).

Therefore, to fill this gap, this article explores the impact of GHRM practices (i.e., green recruitment and selection, green pay, and reward) and dynamic sustainable capabilities (monitoring and re-construction) on corporate sustainability performance in the manufacturing sector. As there is a paucity of research on the causal relationship between GHRM practices, dynamic capabilities, and corporate sustainability performance, this study is timely in filling a clear research gap by employing dynamic resource-based theory and dynamic capability literature. The rationale for incorporating this theoretical lens is to advance both the dynamic research-based view (RBV) and the dynamic capability approach in the context of large-scale manufacturing. This theoretical lens explains how organizational green human resources and sustainable capabilities create a synergic effect to improve the ecological and social performance of the sector. Dynamic RBV provides a guiding paradigm for leveraging dynamic capability framework to better understand, predict and control HR-related practices and ensure continuous resource regeneration within an organization (Helfat and Peteraf, 2003; Singh et al., 2020).

This research makes a significant contribution in terms of theory, method, and practice. Firstly, it theoretically underpins the crux of dynamic RBV theory in the relationship among capabilities, GHRM practices, and corporate sustainability performance to satisfy the demands of the environment and society. The second contribution is the addition of empirical evidence in the scarce literature concerning the manufacturing sector of developing economies. Third, the study employs multisource sampling through well-defined sample selection criteria to prevent common method bias in the model (Podsakoff et al., 2003). Additionally, a large sample size is taken in a developing country context to improve the generalizability of the findings. Also, it validates the recently developed social sustainability scale (Shang et al., 2020) in Pakistani large scale manufacturing industries including textile, automobile, food, and beverages, pharmaceutical and chemical. Further, the study offers an empirical explanation of how green HRM and organizational sustainability-oriented capabilities are critical for corporate performance. Finally, it offers practical implications for the aforementioned industries in the light of the Environmental Protection Agency’s objective of capacity building of stakeholders for better environmental management (Ministry of Environment, 2005) and sustainable workplace practices in the manufacturing sector.

The structure and flow of research include a brief review of GHRM, corporate sustainable performance, and sustainable capabilities from the perspective of the aforementioned theories. The literature review includes research hypotheses and provides gaps and inconsistencies in the previous body of knowledge. Next, research design and methods are discussed, followed by an analysis and in-depth discussion of the findings. Finally, pertinent implications, limitations, and future directions are deliberated.

## Literature Review

### Sustainability as the Dynamic Organizational Capability

Dynamic means changing, evolving in response to or in anticipation of environmental change, and capability is defined as an ability to learn and improve in contrast to an organizational capacity which means holding, accommodating, or receiving knowledge in a restrictive manner. Therefore, Bhupendra and Sangle (2015) define dynamic capabilities as the routines of tasks that when followed are internalized and in the long run become part of organizational capabilities. This is because dynamic capabilities create, combine, and use their resources in new ways; meaning they can synthesize and integrate such routines in unique ways leading to the creation of new knowledge, solution, or configurations (Winter, 2003; Barreto, 2010). So, for example, dynamic capabilities could be adopting new technologies to prevent environmental pollution which will also lead to a competitive edge in the ecological domain. However, for such technologies to flourish, certain micro-level capabilities are required for instance management’s eco-friendly perspective, organizational learning, shared vision, and knowledge through cross-functional integration (Leonidou et al., 2015). Few studies have explored how sustainability can become an organizational capability, enhancing the organizational capacity to mold and innovate toward a sustainable paradigm (Gabler et al., 2015). Also, the resource-based theory is employed by many researchers to understand the organizational response to confront environmental imperatives, However, the dynamic capability framework is better suited for the ever-changing micro and microenvironment of the organization.

However, the work on dynamic capabilities as a dominant perspective in the development of corporate sustainability is scarcely discussed in the literature. Its in-depth discussion is required since such studies can guide corporations to develop required capabilities that can help them to respond successfully to a sustainability challenge arising from their external or internal environment by adjusting their strategies accordingly.

### Typologies of Dynamic Capabilities

According to Teece (2007), there are three coherent clusters of dynamic capabilities including sensing, seizing, and reconfiguration capabilities. Sensing is the capability that identifies and assesses opportunities for sustainability. The process of mobilizing internal and external resources and competencies to capture value is termed seizing capability. Finally, the continuous resource regeneration and orchestration for aligning the organization’s resources with the evolving business environment is referred to as reconfiguration capability. However, another core capability as discussed by Schreyögg and Kliesch-Eberl (2007) is the monitoring capability which refers to continuously checking the validity of capability in regular intervals to prevent it from being obsolete, and facilitating the organization in gaining flexibility and adaptability. Furthermore, the dynamic sustainable capabilities are supported by micro-foundations like employee skills and competencies, organizational routines, and structure which should be considered as a multidimensional construct. These organizational routines are different ways through which the organization can deploy these sensing, seizing, monitoring, and reconfiguration capabilities.

In terms of theoretical approaches employed to study organizational capabilities, the RBV was the most common theory utilized to study resources, capabilities, and strategic management (Barney et al., 2001). RBV provided an explanation regarding the superior performance of some organizations compared to others. Also, Teece et al. (1997) discussed the dynamic capabilities approach to explicate the organizational competitive advantage in dynamic markets. However, focusing on the internal environment only and excluding the ever-changing external environment proved to be a limitation of Resource-Based Theory. This was addressed by Hart (1995) as he proposed the Natural Resource-Based Theory (NRBV) to include natural and organizational resources and environmental issues in RBV. When considering that these resources are ever-evolving, the NRBV transitions to a dynamic resource-based view. In Hart’s view, challenges caused by the natural environment are among the most influential factors in the new pattern of resource development and Organizational Capabilities (Hart and Dowell, 2011). Moreover, over the years, the economic and social environmental challenges raised in the NRBV have multiplied.

In this regard, it is argued by Annunziata et al. (2018) that sustainability-oriented organizations should identify and develop specific capabilities to enhance their competitive advantage. The literature mentions several strategies like the adoption of proactive environmental strategies, for example, Delmas (2011), successful implementation of environmental management systems and practices (Yu and Ramanathan, 2016; Johnson, 2017; Charan and Murty, 2018), adoption of Corporate Social Responsibility (Choi et al., 2019) and management of green supply chain. Hence, it is pertinent to develop the dynamic capabilities to implement these strategies. Despite the fact that there is a body of knowledge on dynamic sustainable capabilities, there is a recent need for more research in this area. According to Gelhard and Von Delft (2016) the literature has been debating about performance in facing economic, social, and environmental issues.

### Organizational Capabilities and Corporate Sustainability

Also, organizations are increasingly considering the environmental issues in their macro-environment which might be the side effect of their production processes on account of tighter regulations by the government or increasing pressure from a wide variety of stakeholders. The literature regarding environmental management is plentiful but not much is known about the organization-centric capabilities which can facilitate to adopt of sustainable environmental management (Grewatsch and Kleindienst, 2018). It was still unclear whether being green is the antecedent of organizational capabilities or whether organizations build certain necessary capabilities which help them to become greener as a consequence. The literature suggests that not only does environmental management increase environmental as well as economic and social performance. Although there is ample literature on environmental management and corporate performance, the role of dynamic sustainable capabilities in explaining their relationship is lacking. Also, there is a lack of integration of social and environmental issues in the theoretical framework and empirical model of previous studies (for instance, Grewatsch and Kleindienst, 2018). In this regard, the importance of corporate sustainability is achieving prominence among organizations. Research has recently studied corporate sustainability from the strategic perspective highlighting that organization’s dynamic sustainable capabilities can have a positive impact on corporate performance (Bocken and Geradts, 2020).

Nevertheless, the relationship between organizational capabilities and sustainability benefits like increased triple bottom line performance remains little explored. For example, Gabler et al. (2015) argue that little is known about how firms determine and use the appropriate resources to maximize the performance of environmental initiatives. Then, the impact of corporate capabilities on corporate performance is addressed by Huang and Huang (2020) but it is limited to the transportation industry and the dynamic nature of internal capabilities is not discussed. Therefore, this study argues the following:

**H1:** Corporate Sustainable Capabilities significantly impact Corporate sustainable performance.
Ha1a: Monitoring Capability has a significant impact on Corporate Social Sustainability Performance.
Ha1b: Monitoring Capability has a significant impact on Corporate Environmental Sustainability Performance.
Ha1c: Reconfiguration Capability has a significant impact on Corporate Social Sustainability Performance.
Ha1d: Reconfiguration Capability has a significant impact on Corporate Environmental Sustainability Performance.

### Green Human Resource Management

Then, according to Jabbour and Santos (2008) and Iqbal (2018), human resource forms the knowledge base of an organization motivating them to invest further into its people. The greening of human resources refers to the set of policies and bundle of practices that enable the organizational human capital to preserve the copious knowledge resource. It is achieved in an eco-friendly and efficient manner in terms of high productivity and low cost (Masri and Jaaron, 2017; Tang et al., 2018). Greening involves engaging in sustainable practices which apply to all the Human Resource functions like recruitment, selection, performance management, pay and reward, employee training, and development among others (Mustapha et al., 2017). It is done to allocate the scarce resources efficiently meanwhile promoting employee morale and their satisfaction with the job which is precisely environmentalism (Renwick et al., 2013; Zaid et al., 2018).

For instance, through recruitment and selection, the potential candidates for employment are informed about the organizational mission and values for sustainability. The green element is included in their job description as well to highlight the desirability of green practices for the organization. Through employee training and development function, green competencies and skills among the employees are fostered. Green team building, green skills, and learning management systems are established to develop and re-enforce pro-environmental behavior (Hameed et al., 2019, 2020). Then, another function of human resource is motivating employee which can be partly done through green performance management which reward employees based on their actions and behaviors about green issues like responsible resource consumption and similar attitude toward the environment and appropriate reward system.

Furthermore, regarding rewards, intrinsic, public, social, and personalized reward systems along with positive performance appraisals lead to pro-social and proactive behavior among employees resulting in higher sustainability performance (Govindarajulu and Daily, 2004; Jackson and Seo, 2010).

The relation between the GHRM and environmental commitment is discussed by Maung et al. (2016) while GHRM’s impact on environmental performance is highlighted by Daily et al. (2012), Kim et al. (2019), and Haldorai et al. (2022). However, the social pillar of the triple bottom line has been mostly ignored in the literature. This scenario is improving since some studies like the one conducted by Amrutha and Geetha (2019) attempt to bridge this gap between GHRM and the social sustainability of the organizations by including the mediating role of Employee Green Behavior (also used interchangeably with the term of Employee Pro-Environment Behavior) and establishing these links through the theories of Ability, Motivation, Opportunity, and Social Identity. Thus there is a major gap in the literature for exploring the social aspect of sustainability in GHRM literature. The questions like what is the contribution of GHRM practices in achieving the triple bottom line can be further explored by empirical testing of each of the Green Human Resource Practice on health, wellness, and wellbeing of the employees along with the organization’s environmental and financial performance.

Also, among GHRM practices, Khan et al. (2020) found that green assessment and rewards are not effective individually for explaining sustainable performance in the case of the Malaysian manufacturing industry. To test their findings, the following is argued:

**H2:** Green human resource management significantly impacts corporate sustainable performance.
Ha2a: Green recruitment and selection have a significant impact on corporate social sustainability Performance.
Ha2b: Green recruitment and selection have a significant impact on corporate environmental sustainability performance.
Ha2c: Green pay and reward have a significant impact on corporate social sustainability performance.
Ha2d: Green pay and reward have a significant impact on corporate environmental sustainability performance.

### Literature Gap

Literature on green HRM and sustainability is limited and shows mixed results in the research (Shafaei et al., 2020; Yong et al., 2020). Further, little discussion is made regarding Green HRM practices leading to corporate sustainability within Pakistan (e.g., Awan et al., 2017). The contribution of Green HRM practices in developing a sustainable working environment has been confirmed by researchers like Chams and García-Blandón (2019) and Muisyo et al. (2021). Yet, the association between green HRM and a firm’s ecological performance has overall mixed findings. For instance, Shahzad et al. (2020) discussed the association of Environment Sustainability to Corporate Social Responsibility and Green Innovation in the Pakistani manufacturing industry. Similarly, Abbas (2020) established a positive relationship between Corporate Social Responsibility with corporate green performance in Pakistani manufacturing industries. But these researchers have considered only one facet of corporate sustainability which is the environment. The other dimensions of sustainability have been largely ignored in the literature like society or social sustainability in a particular sector. The past studies have contemplated the importance of integrating the studies of HRM practices and sustainability-related performance of the organization and it is also suggested for future studies by Macke and Genari (2019) to determine the impact of sustainable HRM practices on the psychological and social wellbeing of individuals. It then manifests in the behavior and commitment of the management.

**FIGURE 1:**
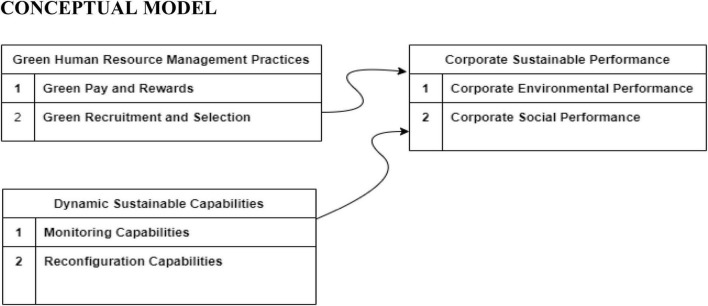
Conceptual framework (author’s development). Inspiration is taken from Amrutha and Geetha (2019) and Nisa et al. (2019).

Moreover, a recent study conducted by Rehman et al. (2021) on 244 Malaysian large manufacturing corporations revealed the lack of association between GHRM and sustainability performance. Consequently, researchers like De Stefano et al. (2018). acknowledge the importance of further examining the relationship between GHRM and corporate social sustainability performance. The paucity of research in this area serves as the motivation to address sustainability performance in the current study seeking empirical evidence of a significant association between GHRM and sustainable performance as conceptualized in Figure 1.

## Materials and Methods

### Data Collection

In terms of the target population, discussion regarding Pakistan’s manufacturing sector is paramount since it is the second-largest contributor to the country’s GDP, the first being the agriculture sector. Therefore, the manufacturing company’s sustainability status or performance is pivotal to study since it can play a significant role in steering Pakistan on the road toward sustainable development (Ikram et al., 2019). Hence, the impact of two macro-level components of management is investigated on two levels of Corporate sustainability performance. Corporate Sustainable Performance, as a result, leads to achieving a competitive edge through balancing improved performance as well as demonstrating a responsible attitude toward society and the environment. This research investigates the relationship between the GHRM, dynamic sustainable capabilities, and corporate sustainability performance variables with each other to test a predictive Corporate Sustainability Performance framework from the perspective of human resource management. Corporate Sustainable Performance has the potential to provide a competitive edge to a highly lucrative industry in Pakistan.

Further, SECP registered and PSX listed organizations from the manufacturing sector make the sampling frame for this research. Six large-scale industries based on their contribution to the GDP, their export percentages, and their contribution to the employment generation are selected and purpose sampling is done (Economic Survey of Pakistan, 2019; OECD, 2020). For instance, textile has the highest contribution to the country’s economy, and the COVID-19 pandemic has presented a unique opportunity; due to the shutdown of China, which was the single largest textile-related exporter, the Pakistani textile industry is working at full capacity to replace China in the global market. Also, it has nearly achieved its export target of $24–25 billion (Siddique, 2020).

The respondents were employees from large-scale manufacturing companies including men and women with 2 years and above experience. These companies belonged to industries like textiles, pharmaceuticals, automobiles, chemicals, food and beverages, and coke and petroleum products from major cities of Pakistan including Karachi, Hyderabad, Lahore, Faisalabad, and Quetta.

### Measurement Development

**FIGURE 2:**
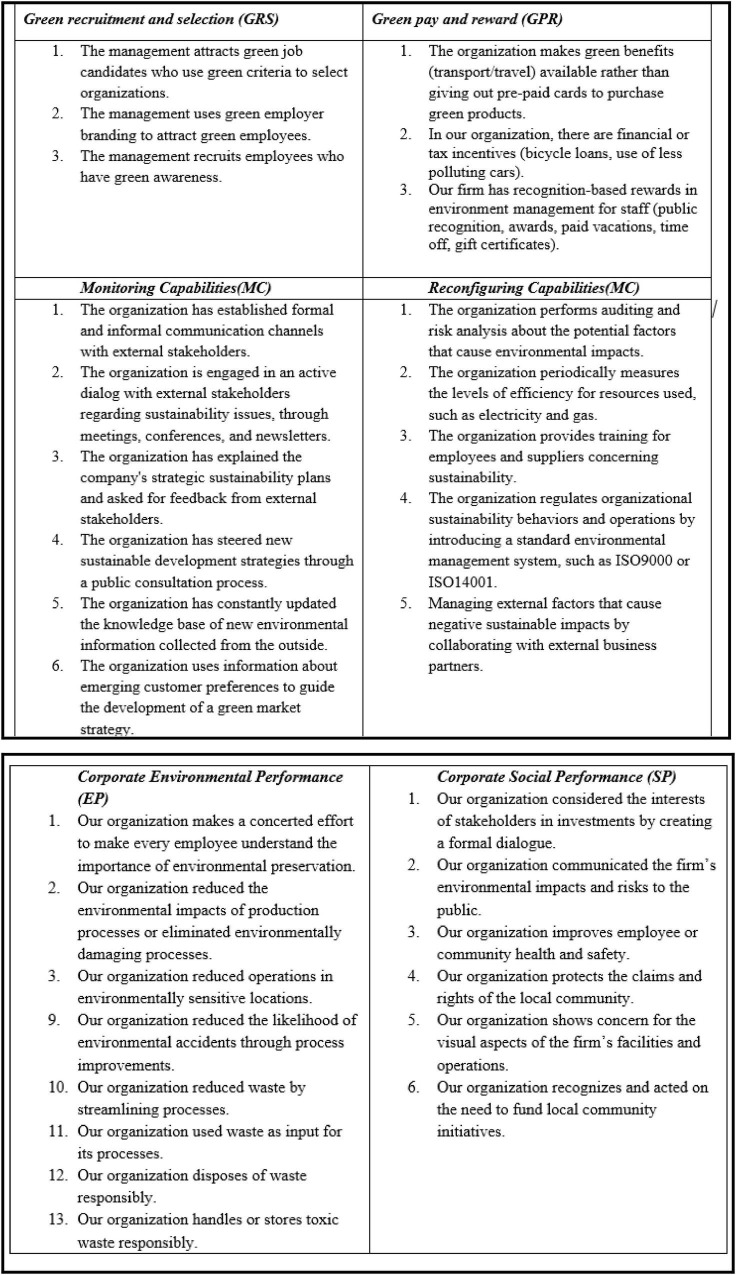
Questionnaire items.

In this study, the items used in the survey were adapted from existing research to fit the context of Corporate sustainable performance and a seven-point Likert scale is used ranging from strongly disagree to strongly agree. Items for GHRM are adapted from the study by Tang et al. (2018). The dynamic sustainable capabilities scale is adapted from Wang and Ahmed (2007) and Shang et al. (2020). Finally, Corporate sustainable performance is defined as the performance of a corporation in all the dimensions or parameters of sustainability including environmental, social, and economic (Schaltegger and Wagner, 2006). Scale adapted from Wijethilake (2017) and Shang et al. (2020). The questionnaire items are shown in Figure 2.

### Data Analysis

#### Respondent’s Demographic Profile

The respondent’s demographical profile was assessed through frequencies and percentage-wise distribution of the pertinent data demonstrated in Table 1. The table provides important insights regarding the respondents and the industry.

**TABLE 1:** Respondent and industry profile.

Variable	Category	Frequency	Percentage
Age	Under 30	225	56.8
	31–40	149	37.6
	41–50	20	5.1
	Above 50	2	0.2
Total		396	100
Education	Undergraduate	80	20.2
	Graduate	280	70.7
	Post-graduate degree	36	10.1
Total		396	100
Marital status	Married	230	58
	Single	166	42
Total		396	100
Gender	Male	338	85.4
	Female	85	14.6
Total		396	100
Management level	Top-level	33	8.3
	Middle level	264	66.4
	Lower level	100	25.1
Total		396	100
Industry	Textile	72	18.2
	Pharmaceuticals	83	21.0
	Food and beverages	83	21.0
	Coke and petroleum products	52	13.1
	Chemicals	63	15.9
	Automobiles	43	10.9
Total		396	100

## Results

### Measurement Model

The relationship between latent constructs and their indicators is examined through the measurement model and it is commonly employed to determine the inter-relationship patterns among the constructs of a conceptual model. To analyze and establish the causal associations among several constructs, a good measurement model is required. The first step in the analysis process is to check the reliability and validity of a measurement model before conducting the tests for the structural model. The measurement model needs to have acceptable levels of reliability and validity.

### Reliability Analysis

The reliability is equal to the squared correlation between the true construct and the construct scores in absence of systematic error. In this research, three types of reliability measures are determined. According to Dijkstra and Henseler (2015), the most important among them is ρA. Then, composite reliability (ρc) and Cronbach’s alpha are also calculated over the measure of composite reliability Jöreskog’s ρ, ω, or ρc, and Cronbach’s alpha is also calculated. In particular, the true reliability is under-estimated by Cronbach’s alpha and therefore, should only be considered as a lower boundary or minimum criteria of the reliability.

Table 2 shows all three reliabilities of the constructs used in this research. All the values of Dijkstra–Henseler’s rho (ρ_*A*_), Jöreskog’s rho (ρ_*c*_), and Cronbach’s alpha exceed the threshold level of 0.7, which represent that the constructs adapted for this research are highly reliable (Henseler et al., 2014).

**TABLE 2:** Reliability analysis.

Construct	Dijkstra Henseler’s rho (ρ_*A*_)	Jöreskog’s rho (ρ_*c*_)	Cronbach’s alpha (α)
Green human resource Mgt-GPR	0.750	0.853	0.742
Green human resource Mgt-GRS	0.835	0.891	0.819
Dynamic sustainable capabilities-MC	0.775	0.850	0.772
Dynamic sustainable capabilities-RC	0.776	0.829	0.733
Corporate sustainable performance-ENP	0.927	0.931	0.913
Corporate sustainable performance-SP	0.941	0.950	0.937

### Validity

Convergent validity is calculated for the constructs to ensure that the items of the same construct should be related to each other as well as the construct. On the other hand, discriminant validity helps to ensure whether items from different constructs are different from each other. The average variance extracted (AVE) is the most common measure of convergent validity whereas the Hetero-trait mono-trait ratio of correlations (HTMT) measures the discriminant validity.

Table 3 exhibits that the convergent validity of each construct exceeds the standard value, i.e., 0.5, confirming the validity of the constructs in the instrument.

**TABLE 3:** Average variance extracted-convergent validity.

Construct	The average variance extracted (AVE)
Green human resource Mgt-GPR	0.659
Green human resource Mgt-GRS	0.732
Dynamic sustainable capabilities-MC	0.596
Dynamic sustainable capabilities-RC	0.551
Corporate sustainable performance-ENP	0.658
Corporate sustainable performance-SP	0.761

#### Hetero-Trait Mono-Trait Ratio of Correlations

**FIGURE 3:**
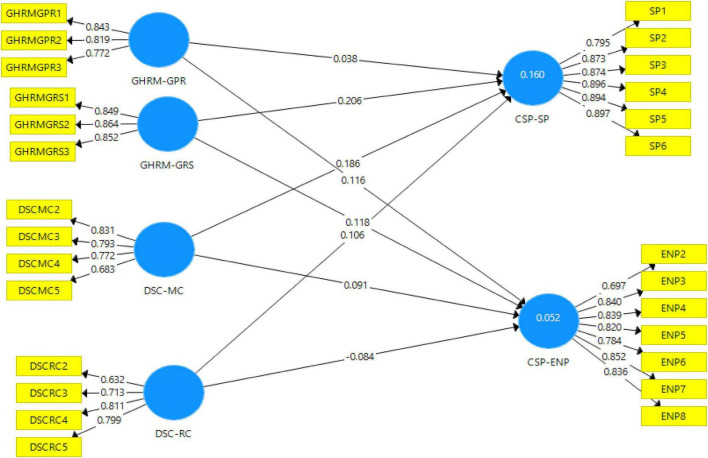
Model-partial least squares (PLS) quality criteria overview.

Moreover, the assessment of discriminant validity is a must in any research that involves latent variables for the prevention of multi-collinearity issues. The HTMT ratio of correlations method is applied in this study, and its threshold value is lesser than 0.90 as depicted in Table 4 (Hamid et al., 2017). The smaller the HTMT of a pair of constructs, the more likely they are to be distinct which is the case in this study’s constructs. The outer model is shown in Figure 3.

**TABLE 4:** HTMT ratio–Discriminant validity.

	CSP-ENP	CSP-SP	DSC-MC	DSC-RC	GHRM-GPR	GHRM-GRS
CSP-ENP						
CSP-SP	0.398					
DSC-MC	0.179	0.371				
DSC-RC	0.094	0.288	0.530			
GHRM-GPR	0.202	0.295	0.674	0.560		
GHRM-GRS	0.185	0.349	0.440	0.325	0.457	

### Outer Model Assessment

Since the reliability statistics are high, therefore, item if the deleted option is not availed in this case. The next step is to move the pertinent data to SmartPLS for predictive model measurement and analysis. The structural model (representing relationships among constructs) has three constructs. Among them, the latent variables were GHRM Practices (GRS and GPR), Dynamic sustainable capabilities (MC and RC), and Corporate sustainable performance (ENP and SP). Based on the hypotheses generated in the previous phase, all the constructs are treated as first-order and reflective in nature (Hair et al., 2017; Sarstedt et al., 2019). The measurement model has twenty-seven indicators, where all items were directly measured in the research sample (reflective in which the construct defines the indicator variables) as conducted by Annunziata et al. (2018) and Zaid et al. (2018) for CSP. Also, organizational capabilities have reflective measurements similar to the study conducted by Huang and Huang (2020). Table 5 shows the outer loadings of the measurement model.

**TABLE 5:** Outer loadings.

Construct	CSP	DSC	GHRM
ENP2	0.697		
ENP3	0.840		
ENP4	0.839		
ENP5	0.820		
ENP6	0.784		
ENP7	0.852		
ENP8	0.836		
SP1	0.795		
SP2	0.873		
SP3	0.874		
SP4	0.896		
SP5	0.894		
SP6	0.897		
DSCMC2		0.831	
DSCMC3		0.793	
DSCMC4		0.772	
DSCMC5		0.683	
DSCRC2		0.632	
DSCRC3		0.713	
DSCRC4		0.811	
DSCRC5		0.799	
GHRMGPR1			0.843
GHRMGPR2			0.819
GHRMGPR3			0.772
GHRMGRS1			0.849
GHRMGRS1			0.864
GHRMGRS3			0.852

#### Assessment of Structural Model

**FIGURE 4:**
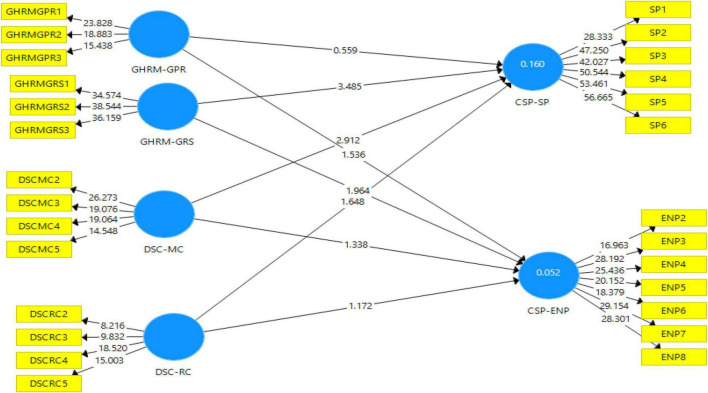
Structural model after bootstrapping.

After testing the outer model, structural analysis (inner model assessment) was conducted with standard assessment criteria which include reporting coefficient of determination (*R*^2^), blindfolding based cross-validated redundancy measure *Q*^2^, and path co-efficient relevance and statistical significance. Figure 4 depicts the structural model after bootstrapping.

### Coefficient of Determination (*R*^2^)

**FIGURE 5:**
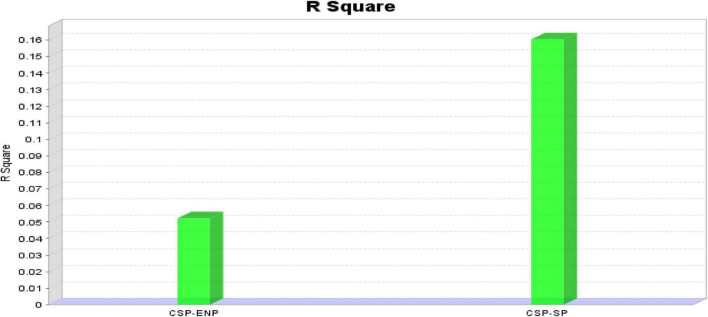
Graphical representation of *R* squared value.

The paths between constructs are exhibited as standardized coefficients. In Table 6, there are two target constructs also known as endogenous constructs whereas the rest of the constructs in the model are categorized as predictors or exogenous. The graphical representation of *R* squared is provided in Figure 5. As suggested by Cohen (1992) in social science research, *R*-square value of 0.12 or below indicates low, between 0.13 and 0.25 values indicate medium, 0.26 or above, and above values indicate high effect size.

**TABLE 6:** The *R* square value.

Predictor construct	Target construct	*R*-squared	*R* square (Adjusted)	*T*-statistics	Predictive accuracy
GHRM, DSC	CSP-ENP	0.052	0.043	2.076	Weak
GHRM, DSC	CSP-SP	0.160	0.152	4.662	Moderate

The *R* square for Corporate sustainable performance is 15.2% meaning 15.2% of the variance in CSP can be successfully explained by these exogenous variables, namely, GHRM and Dynamic sustainable capabilities.

### Predictive Relevance *Q*^2^

The *Q* square measure is used to determine the predictive relevance of the reflective construct in the PLS-SEM model. This statistic is highly relevant since this research model is essentially predictive and has all reflective constructs and indicators. In this case, the construct cross-validated redundancy approach is employed (Hair et al., 2014) because of its suitability in its evaluation. It uses components of the structural, and path model as well as anticipated excluded data points with the following results.

As depicted in Table 7, the value for Corporate sustainability performance meets the criteria; it is significant with a value of greater than zero, however, its predictive accuracy is weak.

**TABLE 7:** Construct cross-validated redundancy.

Total	SSO	SSE	*Q*^2^ (=1-SSE/SSO)
CSP-ENP	2765	2680.46	0.031
CSP-SP	2370	2092.80	0.117

Then, effect size or *f* square needs to be measured which provides the contribution of a variable to the predictive relevance of the concerned construct. It is checked to employ the same threshold values, meaning that according to Hair et al. (2017), *f* square values higher than 0.35 is considered as having a large effect size, 0.15–0.35 are medium, whereas value lying between 0.02 and 0.15 is small. As depicted in Table 8, the independent variables’ DSC and GHRM effect size on the dependent variable (CSP) is small.

**TABLE 8:** Effect size (*f*^2^).

Variables	CSP-ENP	CSP-SP	Effect size
DSC-MC → CSP	0.006	0.028	Small
DSC-RC → CSP	0.006	0.011	Small
GHRM-GPR → CSP	0.009	0.001	Small
GHRM-GRS → CSP	0.012	0.042	Small

*Effect sizes can be assessed by small > 0.02, medium > 0.15, and large > 0.35.*

### Path Analysis

**FIGURE 6:**
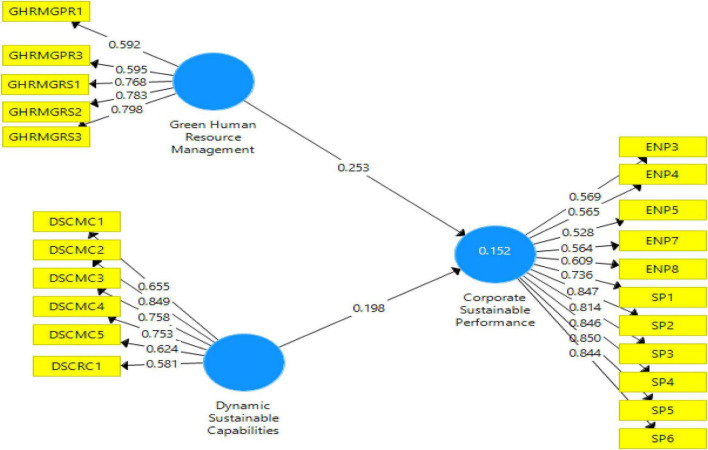
Outer loadings of the main model.

After substantiating the explanatory and predictive powers of our model, the researchers proceeded toward the final steps of testing the extent to which various factors affect Corporate sustainability performance through path analyses. A path model was tested that related two independent variables including GHRM and Dynamic sustainable capabilities with Corporate sustainable performance (Hair et al., 2019). It is shown in Figure 6.

Consistent with Ha1, overall Green HRM was found to significantly impact Corporate sustainable performance CSP (β = 0.198, *p* = 0.00). Hence the first alternate hypothesis was accepted. Ha2 stated that DSC on the whole has a significant impact on Corporate sustainable performance. It was found to be substantiated. (β = 0.257, *p* = 0.00) as shown in Table 9.

**TABLE 9:** Path analyses for the main model.

Hypothesis	Relationship	Original sample	Sample mean	SD	*T*-value	*p*-value	Support
Ha1	DSC → CSP	0.257	0.264	0.056	4.571	0.00	Yes
Ha2	GHRM → CSP	0.198	0.206	0.053	3.748	0.00	Yes

Further, the sub-hypotheses for Ha1 and Ha2 were evaluated in Table 10 with the following result:

**TABLE 10:** Path analysis of the sub-hypotheses.

Hypothesis	Path	Original sample (O)	Sample mean (M)	Standard deviation (STDEV)	*T*-statistics	*p*-values	Support
Ha 1a	DSC-MC → CSP-SP	0.186	0.190	0.064	2.912	0.004	**Yes**
Ha 1b	DSC-MC → CSP-ENP	0.091	0.095	0.068	1.338	0.181	No
Ha 1c	DSC-RC → CSP-SP	0.106	0.112	0.065	1.648	0.099	No
Ha 1d	DSC-RC → CSP-ENP	–0.084	–0.084	0.072	1.172	0.241	No
Ha 2a	GHRM-GRS → CSP-SP	0.206	0.205	0.059	3.485	0.000	**Yes**
Ha 2b	GHRM-GRS → CSP-ENP	0.118	0.118	0.060	1.964	0.050	**Yes**
Ha 2c	GHRM-GPR → CSP-SP	0.038	0.043	0.069	0.559	0.576	No
Ha 2d	GHRM-GPR → CSP-ENP	0.116	0.122	0.075	1.536	0.125	No

The results of the bootstrapping are provided in Table 10 which shows that among the eight hypotheses, three are empirically supported the remaining five hypotheses could not be supported and the hypothesized relationships among the exogenous constructs and with the endogenous construct could not be established for those hypotheses. Moreover, the direct effect has been validated through the two-tailed test with a 0.05 significance level. The above table reveals that green recruitment and selection has a positive and significant impact on both corporate social and environmentally sustainable performance. Further, among the dynamic sustainable capabilities, monitoring capability has a significantly positive impact on corporate sustainable social performance.

### Test for Common Method Bias Through Herman’s Single Factor Analysis

A common problem in research that can compromise its rigor is the presence of common method bias in the research instrument which can become a source of measurement errors (Ardura and Artola, 2020). Extraction sums of squared loadings values below 50% in Table 11 indicate an absence of the common method bias according to Jordan and Troth (2020) and hence add credibility and rigor to our findings.

**TABLE 11:** Test for common method variance.

Table total variance explained	

Factor	Extraction sum of squared loadings (cumulative)
105	20.381

## Findings and Discussion

This research is conducted to determine the impact of GHRM practices and dynamic sustainable capabilities on corporate sustainable performance in the manufacturing sector in a developing country context.

In the first step, descriptive statistics in SPSS were studied which provided insights regarding the manufacturing sector and the respondents’ demographical profiles. On average, there were more male respondents as compared to their female counterparts. Middle and low-level employees were in high percentage as compared to top-level managers who were less than 10% of the total sample. The industries covered included Textile (18.2%), Pharmaceuticals (21%), Food and Beverages (21%), Coke and Petroleum products (13.1%), Chemicals (15.9%), and Automobiles (10.9%).

In the second stage, SmartPLS 3 was employed to assess the outer (measurement) model and inner (structural) model of the research model. Upon running the algorithm, the Cronbach’s alpha, and composite reliability scores were found to be above threshold values. Then convergent validity was assessed through AVE extracted and discriminant validity was found through heterotrait-monotrait (HTMT) ratio and they met the criteria for acceptable values. Any potential Multi-collinearity issues were checked through the Variance Inflation Factor and their values were below 5 as per the conservative estimates. The outer loadings were also found to be greater than 0.5 (Shrestha, 2020). To achieve higher factor loadings some items of the constructs were removed. However, this is not a problem in reflective scales since the items of a construct are inter-changeable. Adjusted *R* square was approximately 15% which means that green human resource management and dynamic sustainable capabilities can explain 15% of the variance in corporate sustainable performance. According to Cohen (1992), *r*-square value 0.12 or below indicates low, between 0.13 and 0.25 values indicate medium, 0.26 and above values indicate high effect size. Therefore, the independent variable has medium explanatory power. Then, the bootstrapping was done to check the *Q* square and *F* square values and both of them indicated low effect size. The path analysis showed that the *t* values higher than 2.5 and *p* values were significant for both main hypotheses.

Therefore, the study found that both GHRM practices and dynamic sustainable capabilities can significantly predict the corporate sustainable performance of the manufacturing sector from a developing country perspective. Similar studies regarding the efficacy of GHRM in the Pakistani manufacturing sector have been conducted by Islam et al. (2020) and Ahmad et al. (2021). Both these studies asserted that corporate ecological performance is improved in the presence of GHRM practices *via* improved employee green behaviors. Furthermore, in the Pakistani manufacturing sector, another study conducted by Islam et al. (2021) proved that Green HRM can mediate the relationship between corporate leadership and employee green behaviors. In particular, green recruitment and selection can promote social and environmentally sustainable performance whereas, monitoring capabilities can successfully predict the social sustainable performance of the sector. Unlike previous findings, the employees do not perceive green pay and reward as predictors of sustainable environmental and social performance. This can be explained by the study of Unsworth et al. (2021) according to which green HRM practices do not work uniformly for all employees. They influence “non-green” employees more as compared to “green” ones. For example, a green feedback and incentives program was successful in engaging some employees but turned off those who already had a strong pro-environmental commitment. Similarly, inadequate evidence of the predictive relationship between reconfiguration capabilities and corporate social and ecological sustainability performance can be explained through the logic provided by Jamal et al. (2021). According to their study, in the fast-paced corporate world, employees have deadlines meant to improve the financial performance of the organization. They are “pushed” to focus more on core activities of daily operations, instead of developing their capabilities in favor of sustainability goals.

Hence, this research is conducted on the environmental and social sustainability performance of a manufacturing sector in a developing country scenario. Similar studies are done by Ghouri et al. (2020), Jayabalan et al. (2020), and Khan et al. (2021) studied Corporate sustainable environment performance in the Malaysian context. According to them, GHRM practices are found to significantly impact sustainable performance which complements the current research. Additionally, they have proposed to include multiple industries from the manufacturing sector in future research. Hence, this suggestion is incorporated and six industries with the highest contribution to GDP are studied in the current study. Also, Masri and Jaaron (2017) found the positive impact of a GHRM practice on environmentally sustainable performance. Then, green selection and recruitment are positively related to sustainable performance. Moreover, our research findings are consistent with Gilal et al. (2019), Khan et al. (2020), and Yusoff et al. (2020) in which GHRM functions such as recruitment and selection positively influence sustainable performance. It furthers the study of Ameer and Khan (2020), by asserting the importance of minimizing environmental and social problems generated due to manufacturing operations. Notwithstanding, further exploration in terms of green HRM in large manufacturing companies in Asia is required.

Then, this research is also consistent with the findings of Mousavi et al. (2018), Bezerra et al. (2019), and Kitenga et al. (2020). These results indicate that there is a significant positive relationship between dynamic capabilities (like sensing, seizing, reconfiguration, and monitoring) and corporate sustainable performance. Therefore, in this manner, our findings support dynamic capabilities theory by confirming that corporate performance and competitive edge are based on organizational ability to respond swiftly to changing external environments.

Finally, to the best of the researchers’ knowledge, this study is the first of its kind to isolate GHRM practices like green recruitment and selection, green pay and reward, and monitoring and reconstruction capabilities to find their impact on corporate social and environmental performance,

## Implications, Recommendations, and Future Research

As asserted by Bombiak and Marciniuk-Kluska (2018) and Yong et al. (2019), GHRM practices and dynamic capability development are a logical solutions for the manufacturing industry to realize the goal of reducing pollution and social value creation. The green value fit between the employer and employee due to green recruitment and selection improve the chances of employees’ involvement in green behaviors on regular basis. It translates into organizations displaying higher corporate social and environmental performance. In addition, it also enhances their satisfaction and retention in the organization as asserted by Pham and Paillé (2019). Based on the findings of the study, it is recommended that the management should focus on GHRM functions, particularly green human resource recruitment and selection, to improve the social and environmental performance of the organization. The idea is to align the recruitment and selection process with the strategic green goals of the organization which leads to the hiring of employees with green values and behaviors.

Moreover, the corporations in which sustainability capabilities are embedded will have a significant inclination to develop monitoring capabilities that are conducive to developing new sustainability-oriented products and processes. Also, as asserted by Marshall et al. (2015), these organizations have a higher propensity to re-define strategy toward social care. Therefore, management should prioritize monitoring capabilities that help in capturing opportunities in the external and internal environment to further their goal of improving the corporate social performance from a sustainability perspective in a developing country context.

From a broader perspective, the findings of the study facilitate corporations in addressing the broad SDG agenda of sustainable consumption and production by adopting GHRM practices. Also, the study makes a case for developing sustainability-oriented capabilities in industries like textiles, pharmaceuticals, food and beverages, chemicals, and automobiles. It furthers Pakistan’s environmental policy aim regarding conserving the country’s environment and improving the quality of life.

This research contributes to the existing literature in the following ways. Firstly, based on the literature gap it attempts to conceptually propose a link between GHRM and dynamic sustainable capabilities with corporate sustainable performance. Secondly, the theoretical gap offered by previous studies with regard to corporate sustainability from an organizational perspective is filled by the current research. In particular, it contributes to determining a pathway between sustainable human resource management attributes and corporate environmental and social performance of firms in the large-scale manufacturing sector. Henceforth, this research is important because it provides a reasonable frame of reference for understanding the dynamics of the sustainable performance of the organizations. It recommends that management should prioritize the acquisition of monitoring capabilities and hiring environmentally conscientious employees by manufacturing firms in their corporate strategy for the attainment of social equity and ecological conservation goals.

Then, the limitations of the study include the fact that this research only focuses on a single sector (manufacturing) and a single country (Pakistan). Also, GHRM practices dynamic sustainable capabilities, and corporate sustainable performance are considered reflective and first-order constructs. For future research, a comparative study can be conducted on corporate sustainable performances in the manufacturing versus service sector. Also, the constructs can be measured as higher-order for future studies.

Further two practices for each independent variable (GHRM and dynamic sustainable capabilities) are considered through the researcher’s discretion for model parsimony. For future studies, the GHRM bundle approach can be adapted to include all GHRM practices for evaluating their impact on corporate sustainability performance. Similarly, for holistic evaluation of this predictive model, Triple Bottom Line Approach can be adopted while assessing the corporate sustainability construct since, in the current study, only social and environmental dimensions are evaluated.

Finally, the COVID-19 pandemic might have influenced the respondents’ perception regarding the sustainable performance-related variables of this research. Therefore, re-collecting the data post-pandemic can improve the predictability of the research model.

## Data Availability Statement

The original contributions presented in this study are included in the article/supplementary material, further inquiries can be directed to the corresponding author/s.

## Author Contributions

MK and NK conceived the manuscript idea and wrote and compiled the major sections of the manuscript. SQ and NK guided regarding the methodology. MK, NK, TH, and AC interpreted research data and critically contributed to the manuscript revisions. All authors contributed to the article and approved the submitted version.

## Conflict of Interest

The authors declare that the research was conducted in the absence of any commercial or financial relationships that could be construed as a potential conflict of interest.

## Publisher’s Note

All claims expressed in this article are solely those of the authors and do not necessarily represent those of their affiliated organizations, or those of the publisher, the editors and the reviewers. Any product that may be evaluated in this article, or claim that may be made by its manufacturer, is not guaranteed or endorsed by the publisher.
